# Exploring the role of gut microbiota in advancing personalized medicine

**DOI:** 10.3389/fmicb.2023.1274925

**Published:** 2023-11-30

**Authors:** Gouxin Huang, Raees Khan, Yilin Zheng, Ping-Chin Lee, Qingnan Li, Imran Khan

**Affiliations:** ^1^Clinical Research Center, Shantou Central Hospital, Shantou, China; ^2^Department of Biological Sciences, National University of Medical Sciences, Rawalpindi, Pakistan; ^3^Biotechnology Research Institute, Universiti Malaysia Sabah, Kota Kinabalu, Sabah, Malaysia; ^4^Faculty of Science and Natural Resources, Universiti Malaysia Sabah, Kota Kinabalu, Sabah, Malaysia; ^5^Department of Pharmacy, Shantou Central Hospital, Shantou, China; ^6^Department of Biotechnology, Faculty of Chemical and Life Sciences, Abdul Wali Khan University Mardan, Mardan, Pakistan

**Keywords:** microbiome, drug, non-response bias, medicine, probiotics, prebiotics

## Abstract

Ongoing extensive research in the field of gut microbiota (GM) has highlighted the crucial role of gut-dwelling microbes in human health. These microbes possess 100 times more genes than the human genome and offer significant biochemical advantages to the host in nutrient and drug absorption, metabolism, and excretion. It is increasingly clear that GM modulates the efficacy and toxicity of drugs, especially those taken orally. In addition, intra-individual variability of GM has been shown to contribute to drug response biases for certain therapeutics. For instance, the efficacy of cyclophosphamide depends on the presence of *Enterococcus hirae* and *Barnesiella intestinihominis* in the host intestine. Conversely, the presence of inappropriate or unwanted gut bacteria can inactivate a drug. For example, dehydroxylase of *Enterococcus faecalis* and *Eggerthella lenta* A2 can metabolize L-dopa before it converts into the active form (dopamine) and crosses the blood–brain barrier to treat Parkinson’s disease patients. Moreover, GM is emerging as a new player in personalized medicine, and various methods are being developed to treat diseases by remodeling patients’ GM composition, such as prebiotic and probiotic interventions, microbiota transplants, and the introduction of synthetic GM. This review aims to highlight how the host’s GM can improve drug efficacy and discuss how an unwanted bug can cause the inactivation of medicine.

## 1 Introduction

Inter-individual heterogeneity in drug response has long been recognized as a major issue that can compromise drug efficacy and harm patients, while also increasing economic burdens. According to a report (Spear et al., 2001) response rates to common drugs for treating human diseases are typically within the range of 50–75%. Various factors have been identified that can affect drug effects, including age, disease condition, concomitant therapy, genome, organ function, and drug interactions (McCallum et al., 2014). However, these factors only partially explain the variability in drug response. In recent years, gut microbiota (GM) has emerged as a new player that significantly affects drug efficacy, particularly for orally-taken medicines that undergo extensive structural modifications by GM enzymes (Weersma et al., 2020). To date, over 200 drugs are known to be metabolized by GM, although the exact species responsible for drug metabolism are often unclear (Spanogiannopoulos et al., 2016). The interaction between drugs and GM is complex, and not limited to antibiotics. Other drugs can also have consequences for GM composition and diversity (Weersma et al., 2020).

The metabolism of drugs by microbes is often carried out through the process of reduction or hydrolysis, but the enzymes that facilitate these processes are largely unknown. With the growing understanding of the importance of GM in the host’s life, several new research fields have emerged, among which is Pharmacomicrobiomics. This field provides a platform to determine the microbial implications in drugs metabolism, response, and disposition (Rizkallah et al., 2010; Bisanz et al., 2018). It is becoming increasingly evident that GM possesses the ability to alter the pharmacodynamics of a medicine by restructuring the medicine or by altering the host’s immunity or metabolism.

According to a recent study, nearly 10^14^ bacterial cells from more than 10,000 different species inhabit the human gastrointestinal tract, which is nearly 10 times the number of host’s cells. These microbes possess 100 times more genes compared to the human genome and offer myriad biochemical advantages in assisting the host with nutrient absorption, metabolism, excretion, and the catalysis of foods and drugs (Sender et al., 2016a,b).

Given the microbial complexity of the gut microbiota and the genetic potential it holds, it is reasonable to infer that it may influence the efficacy of various drugs in various ways. It is now evident that variation in gut microbiota structure has a significant effect on the efficacy of drugs and their associated side effects (Lam et al., 2019). However, research related to how the overall microbiome affects the efficacy of various drugs is still in its infancy, and there are several questions that need to be answered. To uncover mechanistic insights, various computational and wet-lab-based approaches have been utilized (Spanogiannopoulos et al., 2016; Pellock and Redinbo, 2017).

The relative abundance and presence or absence of specific bacterial taxa can increase or decrease the efficacy of drugs. For instance, *Bacteroides* and *Clostridium* spp. have been shown to be correlated with increased activity of the anticancer drug levamisole by metabolizing it to a three-ring-opened metabolite (Shu et al., 1991). Similarly, the genus *Lactobacillus* has also been associated with enhanced drug efficacy. In one study, researchers observed improved efficacy of the anti-retroviral medication tenofovir in a group of patients whose vaginal microbiota was predominated by Lactobacillus. In contrast, weakly responding patients were predominated by *Gardnerella vaginalis*, which was suggested to be responsible for the inactivation of tenofovir (Klatt et al., 2017).

Another example is the non-response bias (NRB) of PD-1 and PD-L1 blockade anticancer therapeutics. The gut microbiota of the responder group was dominated by *A. muciniphila*, *Alistipes* spp., *Eubacterium* spp., and *Ruminococcus* spp. (Matson et al., 2018). Interestingly, the non-responder group showed abundant growth of *B. thetaiotaomicron* and *E. coli* (Gopalakrishnan et al., 2018). It was later confirmed that the number of CCR9+ CXCR3+ CD4+ tumor infiltrating cells increased when *A. muciniphila* was administered with anti-PD1 therapy (Gopalakrishnan et al., 2018; Matson et al., 2018; Routy et al., 2018). These investigations highlight the possibility that alterations in the composition of the GM population can influence therapeutic effectiveness.

Numerous studies have investigated how microbial metabolism affects anti-inflammatory drugs. The conversion of sulfasalazine into its active form by the GM is another example of how the microbiome can influence the efficacy of a drug (Spanogiannopoulos et al., 2016). Integrating GM and drug research could provide new insights into combating diseases and improving human health by developing novel co-therapies, discovering biomarkers and drug targets. The GM could contribute to lowering non-response bias of a drug and enhancing its efficacy by metabolizing medicine or modifying the host’s immune response by secreting immunometabolites and/or presenting specific antigens (Weersma et al., 2020).

This review provides an in-depth examination of the interplay between GM and drugs and their impact on patients’ health. While previous reviews have focused on how the microbiome affects our response to drugs and the potential for personalized medicine, this review offers new insights into the role of the host microbiome in drug interactions. Specifically, we highlight how the host microbiome can potentially minimize non-response bias of psychiatric, anti-diabetes, anti-cancer, antihypertensive, and anti-arthritic drugs. Furthermore, we discuss novel potential strategies for microbiome manipulation, including functional bioprospecting metagenomics, customized SynCom-based microbiota transplants, and the use of bacteriophages. By exploring these new approaches, we aim to provide a comprehensive understanding of how GM and drugs interact and how we can harness this knowledge to optimize patient outcomes. The article also discusses the impact of GM on drug metabolism, effectiveness, and safety. The specific genes encoded by GM that can metabolize drugs and affect drug effects are highlighted using examples of cardiovascular drugs digoxin and diltiazem, as well as nifedipine, a drug used to treat precordial angina and hypertension. The article also mentions the promotion of beneficial bacteria growth after administering medicine to hosts and highlights the growing interest in finding out how certain drugs could selectively increase the growth of specific bacteria.

## 2 GM–drug interaction

### 2.1 GM metabolize the drugs

GM can affect drug safety and effectiveness by enzymatically changing drug structure, modifying drug availability, and altering bioactivity or toxicity. Digoxin, a cardiovascular drug commonly used for patients with heart failure and atrial fibrillation, is converted to dihydrodigoxin by about 10% of patients, which inactivates the effect of digoxin. *Eggerthella lenta* is responsible for initiating the digoxin inactivation process. *E. lenta* possesses a two-gene cardiac glycoside reductase (*cgr*) operon, which is up-regulated by digoxin. The *cgr* operon encodes two associated proteins, CGR1 and CGR2, which can form a protein complex. CGR2 possesses a digoxin binding pocket that contains several non-polar hydrophobic residues and negatively charged polar amino acids. The active site of the CGR1/CGR2 complex can metabolize digoxin to inactive forms, such as digoxin reduction products and dihydrodigoxin. Another gene encoded by *Bacteroides thetaiotaomicron* has been shown to metabolize drugs, similar to the gene encoded by *E. lenta*. Diltiazem, an oral calcium channel blocker used for patients with hypertension, arrhythmia, and angina pectoris, is metabolized to various metabolites *in vivo* to exert its bioactivities (Boyd et al., 1989). *Zimmermann* et al. combined mass spectrometry with high-throughput genetic sequencing to systematically identify microbial gene products involved in drug metabolism. The results showed that the gene *bt4096*, encoded by *B. thetaiotaomicron*, was a potential target for the metabolism of diltiazem. The authors colonized germ-free mice with either the *B. thetaiotaomicron* wild type or the *bt4096* deletion strain and orally treated them with diltiazem. The quantification of the drug kinetics showed that the deacetylation of both diltiazem and diltiazem metabolites in the gut was *bt4096*-dependent. Moreover, the metabolism of diltiazem by *B. thetaiotaomicron* was enhanced with repeated oral administration of this bacterium (Zimmermann et al., 2019). These studies displayed the specific genes encoded by GM which can metabolize drugs and impact the drug effects.

Nifedipine, a nonpolar drug that is completely absorbed by the gastrointestinal tract, is usually used to treat precordial angina, hypertension, and other vascular diseases (Grigoriev et al., 2016; Filgueira et al., 2017). A study compared the pharmacokinetics and metabolism of nifedipine by different GM compositions among rats exposed to plateau hypoxia, plain, and antibiotic treatment. After 12 h of incubation of nifedipine with the rats’ feces from different groups, the results showed that nifedipine levels decreased by 34.79% in the plateau group, 53.72% in the plain group, and 42.57% in the antibiotic-treated group. Meanwhile, the percentage of metabolized nifedipine was 10.84, 23.14, and 16.67%, respectively. MS/MS analysis results showed that oxidized nifedipine is the main metabolic product by GM. Although the authors found a significant difference in GM composition among these 3 groups, for instance, the relative abundance of *Bacteroidetes* was significantly up-regulated, and *Prevotella* was down-regulated in the plateau group, the underlying mechanism of how GM affects nifedipine metabolism needs further investigation (Zhang et al., 2018). For other drugs in patients with hyperlipidemia, including simvastatin, rosuvastatin, and atorvastatin, several reports showed that GM could affect the drugs’ metabolism and absorption through bacterial-derived bile acids, but the specific bacteria and underlying mechanisms are still unknown (Kaddurah-Daouk et al., 2011; Nolan et al., 2017; Liu et al., 2018). Notably, based on the mass spectrometry and high-throughput genetics sequencing platform, the study showed that 76 diverse GM could metabolize 271 oral drugs, and many of these drugs were chemically modified by GM. Although the exact mechanism by which GM metabolizes drugs is unclear, this study exhibited the key role of GM in the metabolism of drugs, as well as pointing out the targeted genes for the drugs’ metabolism (Zimmermann et al., 2019).

**Figure 1:**
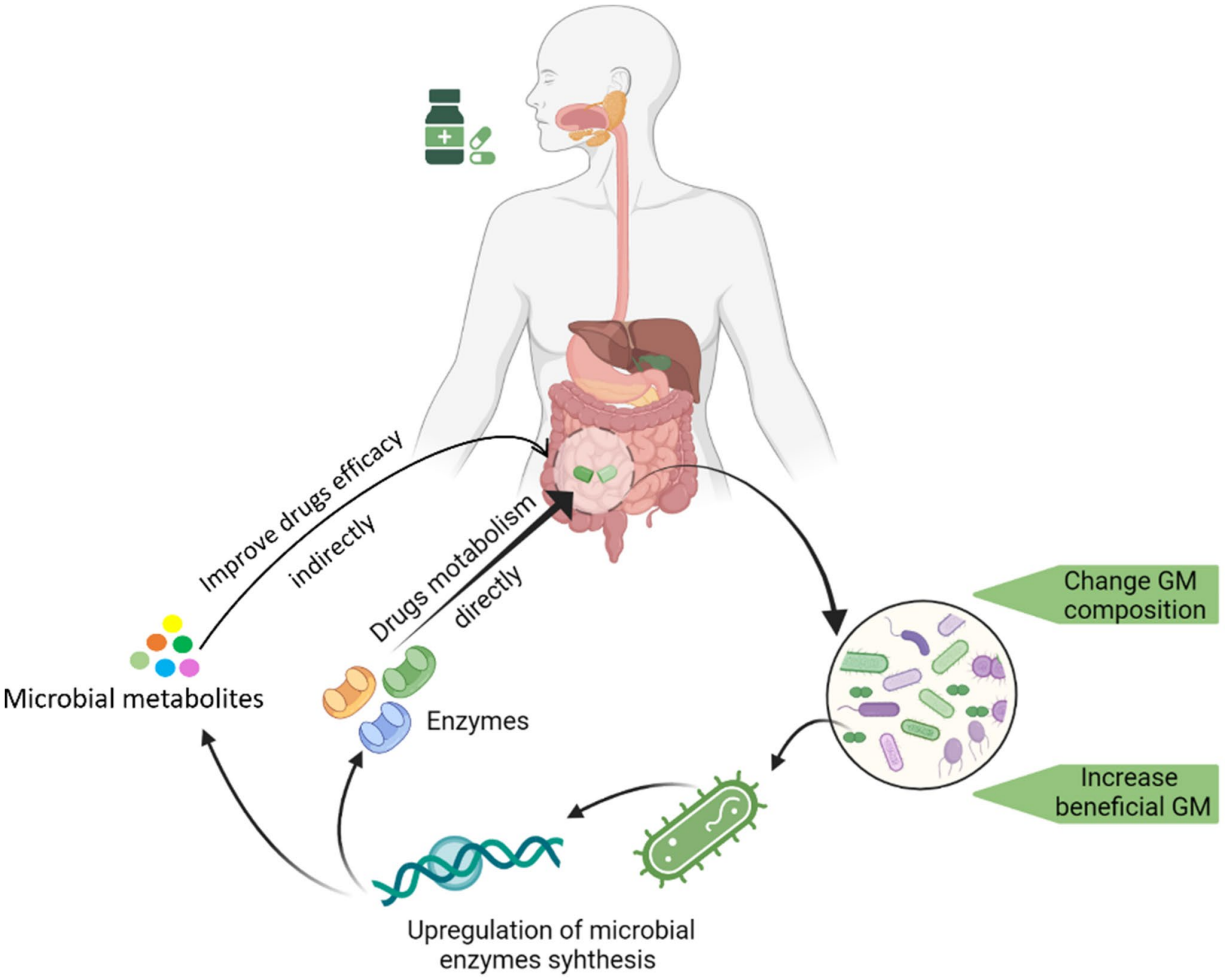
Graphical illustration of the drug-induced changes in GM and the underline changes in the genome of a drug-promoted bacterium enhancement.

Several studies have reported the promotion of beneficial bacteria growth after administering medicine to hosts. There is growing interest in finding out how certain drugs could selectively increase the growth of specific bacteria. In one such attempt, we determined that saponin could promote the growth of *Bifidobacterium animalis* by upregulating several key bacterial genes involved in biogenesis and metabolic pathways (such as *gatC*, *rpmH*, *ruvA*, *yajC*, and *rsfS*) (Liao et al., 2020). We have summarized this whole process by depicting key steps in Figure 1.

### 2.2 GM improve the drugs bioavailability

It is important for oral drugs with high bioavailability to exert therapeutic effects. A number of prodrugs were abandoned in preclinical studies due to their low bioavailability. Gut commensal microbes can interact with drugs after they enter the gastrointestinal tract and affect the drugs’ bioavailability. Some evidence suggests that gut microbes can compete with and regulate drug transporters to affect bioavailability (González-Sarrías et al., 2013). Other reports have shown that GM-derived enzymes can directly modify the bioavailability of oral drugs by regulating drug metabolism, the first-pass effect, and enterohepatic circulation (McCabe et al., 2015; Zhang et al., 2021). In addition to local GM, infection by pathogens and probiotic supplements have also been shown to modulate drug bioavailability (Pierantozzi et al., 2006; Matuskova et al., 2014).

Coating prodrugs with an azo molecule is a strategy for prodrug design because azo reduction can be conducted by azoreductases of GM, releasing the active moiety to exert pharmacological actions (Childs et al., 1967; Zhang et al., 2021). An example of an azo prodrug used for inflammatory bowel disease in clinics is sulfasalazine. According to related reports, only 12% of sulfasalazine was absorbed in the upper gastrointestinal tract. However, the major therapeutic molecule, 5-aminosalicylic acid (5AA), was released after being catalyzed by GM through the azo reduction pathway in the colon (Ray, 2019). To avoid off-site absorption and increase drug bioavailability, 5AA was conjugated with an azo molecule. Drugs such as olsalazine, ipsalazide, and balsalazide were designed using the same strategy (Chan et al., 1983; Truelove, 1988; Zhang et al., 2021). GM, including the genera *Clostridium* and *Eubacterium* and the species *Lactobacillus acidophilus*, *Bifidobacterium lactis*, and *Streptococcus salivarius*, have been shown to have the capacity for azo reduction (Rafii and Cerniglia, 1995; Lee et al., 2012).

The first-pass effect is a crucial factor that mainly impacts drug availability and therapy. Drugs are either metabolized in the gastrointestinal tract or transported to the liver via the portal vein and then metabolized in the liver. These processes can decrease drug concentration in the systemic circulation (Gibaldi et al., 1971). GM-induced first-pass effect has been shown to significantly affect drug pharmacokinetics. Aspirin, a non-steroidal anti-inflammatory drug widely used in clinics for inflammation-associated diseases, can be bio-transformed into salicylic acid in the gastrointestinal tract by GM, reducing serum concentrations of aspirin (Kim et al., 2016). However, treatment with antibiotics can reverse this phenomenon. Sun et al. reported that an increase in *Bacteroides* but a decrease in *Prevotella*, *Corynebacterium*, and *Coprococcus* can improve aspirin absorption in rats (Sun et al., 2020).

Enterohepatic circulation is another physiological process that modifies drug bioavailability. Drugs are catalyzed and bio-transformed in the liver, where they are absorbed, and their metabolites are re-delivered to the intestinal tract via the bile duct. Due to catalyzation by GM, reactivated drugs are reabsorbed by gut epithelia and sent back to the liver (Pellock and Redinbo, 2017; Zhang et al., 2021). β-glucuronidases, secreted by GM, are important enzymes during this process. They catalyze the hydrolysis of glucuronidated drugs formed in the liver by uridine diphosphate-glucuronosyltransferases, releasing and reactivating aglycones in the intestinal tract (Spanogiannopoulos et al., 2016; Pellock and Redinbo, 2017). GM including *Peptostreptococcus*, *Clostridium*, and *Staphylococcus* produce and secrete β-glucuronidases (Gadelle et al., 1985). Indomethacin is also a non-steroidal anti-inflammatory drug, and the associated report showed that mice pretreated with antibiotic cocktails could induce lower serum concentrations and shorter half-life of the drug attributing to the decrease of β-glucuronidases (Liang et al., 2015).

### 2.3 GM influence the efficacy of drugs

Although our understanding of the impact of the GM on drug efficacy is still in its nascent stage, recent research has highlighted its crucial role. The GM plays an important role in the transformation of pharmaceuticals, altering their bioactivities, toxicities, and lifetimes within the body. A better understanding of the relationship between the complexity and diversity of the GM and drug efficacy has the potential to guide personalized medicine and nutrition, inform toxicology risk assessments, and improve drug research and development in pre-clinical and clinical studies (Koppel et al., 2017).

5-fluorouracil (5-FU) and its prodrug, such as capecitabine, are commonly used in cancer therapy, particularly for colorectal cancer. These drugs have been reported to inhibit nucleotide biosynthesis and cell division by inhibiting thymidylate synthase. However, there is no universal dosage of 5-FU in clinical practice, and significant pharmacokinetic variations exist among patients. Additionally, frequent adverse effects, such as diarrhea, nausea, and peripheral blood cytopenia, are observed in clinical settings (Sonis et al., 2004). The response rate of 5-FU is about 10% when used as monotherapy, and about 50% when combined with other chemotherapies (Longley et al., 2003). The inter-patient variation of 5-FU efficacy and toxicity is not solely due to host genetic differences, but also due to differences in GM composition and their unique physiological processes. A study demonstrated that implantation of five different strains of *Escherichia coli* in *C. elegans* resulted in significant differences in 5-FU minimum inhibitory concentration (MIC). The results further showed that variations in 5-FU MIC also existed in different species. Notably, the variation of 5-FU efficacy disappeared when implanted with devitalized bacteria. Based on gene sequencing, the authors found that the pyridoxal-5-phosphate (PLP) biosynthesis pathway was associated with 5-FU efficacy. PLP is the active form of vitamin B6, and *E. coli* possesses the capacity for PLP synthesis via both *de novo* and salvage biosynthetic pathways (Fitzpatrick et al., 2007). Additionally, the inhibition of *de novo* PLP synthesis could significantly reduce the efficacy of 5-FU. Furthermore, the results also demonstrated that the disruption of microbial deoxynucleotide pools could increase 5-FU-induced autophagy and cell death in host cells (Scott et al., 2017).

Levodopa is a medication commonly used for Parkinson’s disease treatment. It is absorbed by the small intestine, crosses the blood–brain barrier, and is converted into dopamine by the human enzyme aromatic amino acid decarboxylase, thus exerting its therapeutic effects. Co-administration of catechol metabolism inhibitors has been shown to increase levodopa efficacy by preventing its off-site metabolism. However, recent studies have revealed that tyrosine decarboxylases (*tyrDCs*) produced by certain bacteria, such as *Enterococcus faecalis* and *Lactobacillus*, can convert levodopa to dopamine before it reaches the brain, significantly reducing its therapeutic benefits in Parkinson’s patients. Interestingly, the same studies have shown that inhibiting microbial *tyrDCs* gene expression can block this conversion and improve levodopa efficacy (Maini Rekdal et al., 2019; van Kessel et al., 2019; Weersma et al., 2020). In addition, another study evaluated the efficacy of 30 different anticancer drugs incubated with either *E. coli* or *Listeria welshimeri* in cancer cell lines *in vitro*. The authors found that *E. coli* could change the drug’s efficacy, e.g., gemcitabine, fludarabine, and CB1954, by modifying the drugs’ structure when co-incubated with these drugs (Lehouritis et al., 2015). These studies suggested that GM affected the drug’s efficacy through the microbial- driven modification of the drugs’ structure.

### 2.4 GM modify the drugs toxicity and adverse effects

Apart from influencing the metabolism, bioavailability, and efficacy of drugs, research has shown that the GM can also modify drug toxicity and adverse effects. For instance, cyclophosphamide, a commonly used anti-cancer chemotherapeutic agent, has marked adverse effects such as villus damage, inflammatory cell recruitment, and gut epithelial barrier dysfunction. Studies have linked these adverse effects to the reduction of the genus *Lactobacilli* and *Enterococci* (Vétizou et al., 2015). Similarly, irinotecan, another anti-cancer drug, can be metabolized to SN-38 by microbial enzyme β-glucuronidases, leading to severe diarrhea in clinical settings (Chamseddine et al., 2019). In one mouse model study, the administration of irinotecan with a β-glucuronidase inhibitor prevented irinotecan-induced diarrhea (Wallace et al., 2010). Additionally, β-glucuronidases can induce high concentrations of non-steroidal anti-inflammatory drug aglycones, which can damage the small intestine’s structure through mitochondrial stress and endoplasmic reticulum stress (Saitta et al., 2014).

Cisplatin is a widely used chemotherapeutic agent for cancer therapy. Joyce et al. (2010) reported that cisplatin possesses an antibiotic effect that inhibits the growth of gut microbiota, such as *E. coli* and *Bacillus*, leading to dysbiosis (Joyce et al., 2010). Cisplatin can also impair the gut epithelial barrier by binding to DNA and inhibiting the proliferation of gut epithelia However, a related study showed that fecal microbiota transplantation (FMT) could resist life-threatening sepsis in patients treated with cisplatin (Taur and Pamer, 2016). Notably, an associated study showed that FMT could resist life-threatening sepsis in patients treated with cisplatin (Perales-Puchalt et al., 2018). Another study showed that D-methionine could prevent cisplatin toxicity by reconstructing dysbiosis through increasing the relative abundance of beneficial bacteria, such as *Lachnospiraceae* and *Lactobacillus* (Wu et al., 2019).

As previously mentioned, intraperitoneal injection of 5-FU often causes mucositis, inflammation, and even bacteremia and sepsis (Sonis et al., 2004; Anke et al., 2017). Several reports have shown that 5-FU leads to dysbiosis, from commensal bacteria (i.e., *Lactobacillus* spp. and *Bifidobacterium*) to dominated genera of *Escherichia*, *Enterococcus*, and *Clostridium* (Hamouda et al., 2017). Emerging evidence has shown that GM dysbiosis can aggravate the toxicity of 5-FU and accelerate the inflammation process (Gori et al., 2019). However, animal models treated with 5-FU have shown that the depletion of gut microbiota by antibiotics cocktails can reduce intestinal mucositis and inflammatory cytokine secretion (Hamouda et al., 2017).

**Figure 2:**
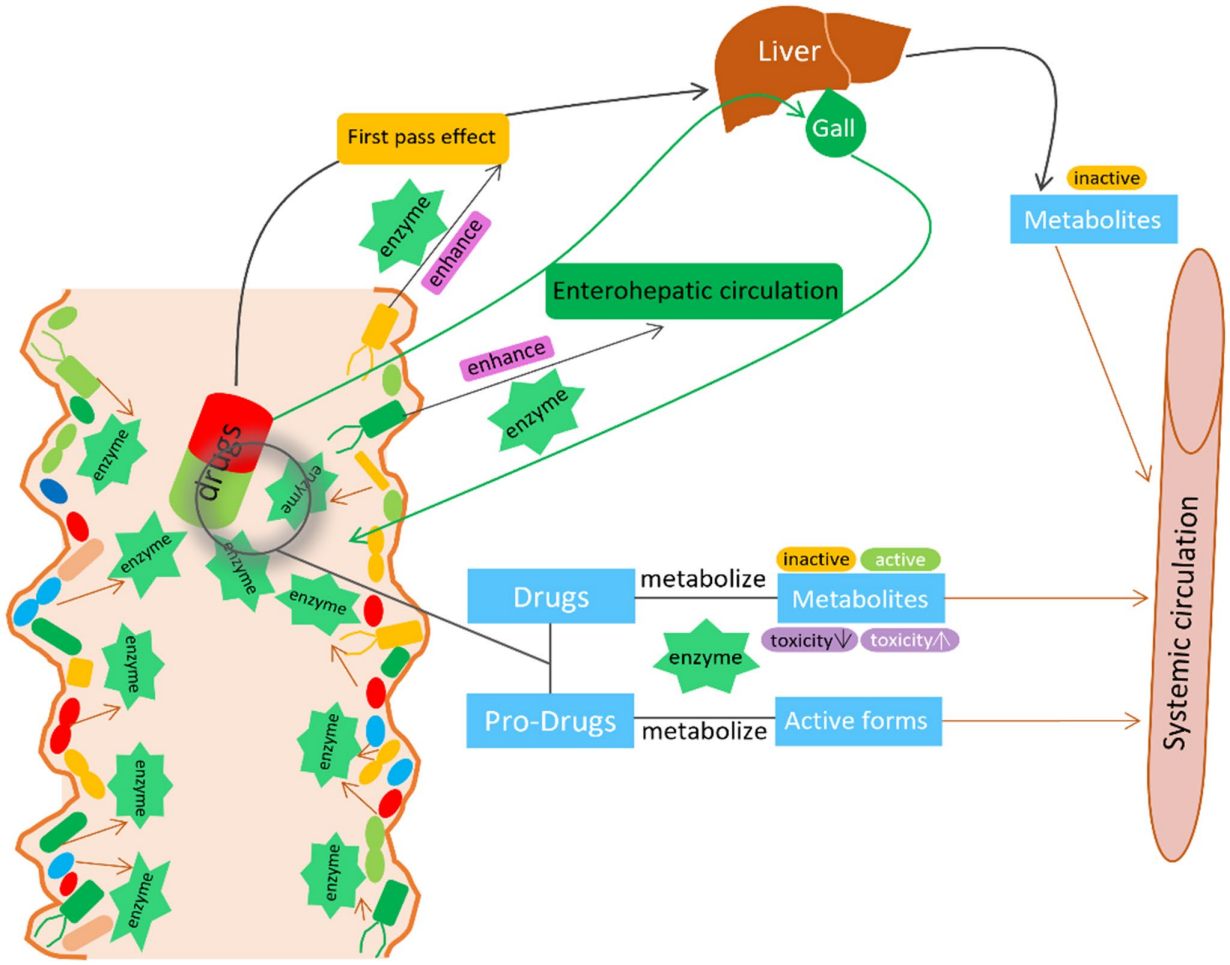
The interaction between GM and drugs. GM activate/inactivate the drug efficacy by the microbial derived enzymes. GM also enhance the first pass effect and enterohepatic circulation by the microbial derived enzymes to modify the metabolism, bioactivity, bioavailability of the drug.

Overall, these studies highlight the important role of gut microbiota in modifying drug adverse effects and toxicity. Therefore, manipulating gut microbiota seems to be a plausible strategy to reduce side effects and toxicity while improving drug efficacy and safety (Table 1 and Figure 2).

**Table 1:** The interaction between GM and the drugs.

Drug	GM	The effect of GM on drugs	Ref.
Digoxin	*Eggerthella lenta*	Metabolized digoxin to the inactive form, e.g., digoxin reduction products and dihydrodigoxin by the encoded gene of cardiac glycoside reductase	Boyd et al. (1989)
Diltiazem	*Bacteroides thetaiotaomicron*	Deacetylated diltiazem by the encoded gene *bt4096*	Zimmermann et al. (2019)
Nifedipine	Different GM from rats fed in plateau hypoxia, plain, and antibiotic treatment	Rats GM from the plateau group could reduce the metabolism of nifedipine in serum when compared to the other 2 groups	Zhang et al. (2018)
Sulfasalazine, lsalazine, Ipsalazide, and Balsalazide	*Clostridium* and *Eubacterium, Lactobacillus acidophilus, Bifidobacterium lactis*, and *Streptococcus salivarius*	Increasing the bioavailability of the drugs by the capacity of the azo reduction which could release the therapeutic molecule, 5-aminosalicylic acid in the colon	Rafii and Cerniglia (1995), Lee et al. (2012), and Ray (2019)
Aspirin	Increasing *Bacteroides* but decresing *Prevotella* and *Corynebacterium*	Increasing the absorption of aspirin by inhibition of the bio-transform of aspirin	Kim et al. (2016) and Sun et al. (2020)
Non-steroidal anti-inflammatory drugs, e.g., Indomethacin	*Peptostreptococcus*, *Clostridium*, and *Staphylococcus*	Enhancing the enterohepatic circulation by β-glucuronidases which catalyzed the hydrolysis of the drugs to modify the drug’s bioactivities	Gadelle et al. (1985), Liang et al. (2015), Spanogiannopoulos et al. (2016), and Pellock and Redinbo (2017)
5-fluorouracil	*Escherichia coli* strains	Increasing the synthesis of pyridoxal-5-phosphatevia to improve the effect of 5-fluorouracil	Fitzpatrick et al. (2007)
Levodopa	*Enterococcus faecalis* and *Lactobacillus*	Convert levodopa to dopamine before levodopa entered the brain by bacteria-driving tyrosine decarboxylases	Maini Rekdal et al. (2019), van Kessel et al. (2019), and Weersma et al. (2020)
Gemcitabine and Fludarabine	*Escherichia coli*	Changed the drugs’ structure when co-incubate with the drugs	Lehouritis et al. (2015)
Cyclophosphamide	*Lactobacilli* and *Enterococci*	Reducing the adverse effect of cyclophosphamide	Vétizou et al. (2015)
Cisplatin	Fecal microbiota transplantation	Resisted life-threatening sepsis in patients treated with cisplatin	Taur and Pamer (2016)
5-fluorouracil	Depleted GM by antibiotics	Reducing the intestinal mucositis and inflammatory cytokines secretion in animals treated with 5-fluorouracil	Hamouda et al. (2017)

## 3 GM can minimize the NRB of drugs

### 3.1 GM can minimize NRB of psychiatric drugs

The microbiota-gut-brain axis, a recently discovered phenomenon, refers to the interaction between the enteric nervous system and the central nervous system through metabolites, hormones, and neurotransmitters (Boyd et al., 1989; Fitzpatrick et al., 2007; Cryan et al., 2019). The vagus nerve (VN) is the longest cranial nerve in the body that can regulates gut physiology. Growing literature supports the actions of GM to modulate gut-brain signaling via VN, for instance, administration of *C. rodentium* could increase anxiety-like behaviors in a vagally-dependent manner (Lyte et al., 2006), while *Lactobacillus* and *Bifidobacterium* strain normalized anxiety-like behavior, again in a manner involving the VN (Bercik et al., 2011; Bravo et al., 2011). Clinical studies also proved that probiotics may decrease anxiety and depression (Dinan and Cryan, 2013). Accumulating research has shown that dysbiosis of the gut microbiome is associated with neurological diseases such as mood disorders, including anxiety and depression, and neurodegeneration, such as Alzheimer’s disease (AD) and Parkinson’s disease (PD) (Mulak and Bonaz, 2015; Dinan and Cryan, 2017). A recent study also showed that intake of antibiotics could induced GM depletion and altered metabolite levels and may affect spleen and brain function through the gut-microbiota-spleen-brain axis (Wan et al., 2023). Some studies have reported that antipsychotics possess antibacterial effects, and the authors suggested that this could be one of the mechanisms of antipsychotics (Mandal et al., 2010; Jena et al., 2014; Maier et al., 2018). There is also a close correlation between the gut microbiome composition and the efficacy of antipsychotics.

Selenium is an important trace element that plays a pivotal role in physiological processes. Studies have shown that selenium can be used to prevent the progression of AD (Hatfield et al., 2014; Zhang Z.-H. et al., 2017). A randomized, double-blind, and controlled trial that enrolled 79 AD patients showed that patients treated with selenium plus probiotics had significantly increased Mini-Mental State Examination scores, total antioxidant capacity, and glutathione compared to those treated with selenium and placebo. In addition, patients treated with selenium plus probiotics showed down-regulation of serum triglycerides, LDL, and total cholesterol compared to those treated with selenium and placebo. The authors claimed that supplementation with probiotics could improve the benefits of selenium for AD patients (Tamtaji et al., 2019).

Curcumin, a major component derived from many herbal medicines, has been shown to possess neuroprotective bioactivities in neurodegenerative diseases. A preclinical study used *Lactobacillus rhamnosus* as a curcumin adjuvant to treat scopolamine-induced dementia in mice. The results showed a prominent increase in antioxidant enzymes in the group receiving curcumin plus *L. rhamnosus* compared to the group receiving only curcumin. Additionally, histological results showed that curcumin plus *L. rhamnosus* could reduce neuronal damage compared to curcumin alone. Moreover, curcumin plus *L. rhamnosus* had a better effect on memory and cognitive improvement. The authors suggested that probiotics could enhance the effect of curcumin in treating AD (Patel et al., 2020).

PD is a progressive neurological disorder characterized by motor and non-motor features. Treatment with dopaminergic drugs is symptomatic and aims to control motor disturbances (Sveinbjornsdottir, 2016). Levodopa is the standard and initial therapy for PD patients; however, with continued treatment and as the disease progresses, up to 80% of PD patients experience “wearing-off” symptoms, dyskinesias, and other complications (Jankovic and Stacy, 2007). These levodopa-associated problems can profoundly affect the quality of life of PD patients. Reports have shown that many gut microbiota possess the gene-encoded tyrosine decarboxylase (TDC), which can produce dopamine from levodopa in the gastrointestinal tract. Consequently, *in situ* levels of levodopa are significantly reduced in patients with intestinal bacterial TDC, thus reducing the drug’s effectiveness through catalytic reactions (van Kessel et al., 2019). However, an *in vitro* study showed that supplementation with *Lactiplantibacillus plantarum* PS128 could improve the effect of levodopa in a 6-hydroxydopamine-induced PD rat model (Ma et al., 2021).

### 3.2 GM can minimize NRB of anti-diabetes drugs

Metformin is an oral blood-glucose-lowering drug used for the treatment of type 2 diabetes (T2D) through multiple mechanisms. It significantly inhibits liver gluconeogenesis and regulates gut microbiota (GM) composition, providing benefits to the treatment of T2D (Shin et al., 2014; Sun et al., 2018). Clinical reports have shown that metformin induces changes in over 80 species of GM when compared to healthy volunteers. Metformin significantly increases the relative abundance of *E. coli* but decreases the genus of *Intestinibacter* (Wu et al., 2017). Notably, reports have also shown that up to one-third of patients treated with metformin experience adverse effects, including diarrhea, nausea, and bloating, which could be attributed to the changes in GM composition (Forslund et al., 2015; Wu et al., 2017). An *in vitro* study using a streptozotocin-induced diabetes rat model showed that metformin and montelukast, in combination with Lactobacillus, could significantly reverse testicular and liver damage and reduce oxidative, inflammatory, and apoptotic activities caused by streptozotocin. Furthermore, the results also showed that metformin and montelukast, combined with Lactobacillus, had a better effect on GM dysbiosis caused by streptozotocin, such as increasing the genera of *Bifidobacteria* and *Lactobacillus*, while decreasing the abundance of *Bacteroides* spp., *Clostridium* spp., *Fusobacterium* spp., and *E. coli* (El-Baz et al., 2021).

Acarbose is an effective and safe medication for T2D which is a glucoamylase inhibitor to prevent starch digestion in the small intestine and lower postprandial blood glucose levels. Reports showed that treatment of acarbose could significantly change GM composition of pre-diabetic patients. The genus of *Lactobacillus* and *Dialister* could be markedly stimulated, whereas, the genus of *Butyricicoccus*, *Phascolarctobacterium*, and *Ruminococcus* were inhibited by the administration of acarbose (Zhang X. et al., 2017). Moreover, a study showed that the effect of acarbose on GM composition was diet-dependent and reversible in the mice model. The result showed that once the treatment of acarbose is stopped, the change of GM community caused by acarbose could rapidly revert to resemble that of the control group (Baxter et al., 2019). The latest study showed that human oral and gut microbiomes encode enzymes that could selectively target and inactivate acarbose through phosphorylation. Using biochemical assays, X-ray crystallography, and metagenomic analyses, the authors clarified that the microbiome-derived acarbose kinases are specific and widespread in the microbiomes of western and non-western human populations (Balaich et al., 2021). This evidence showed that one potential strategy to enhance the effect of acarbose could be via manipulating GM composition.

### 3.3 GM can minimize NRB of anti-cancer drugs

As mentioned earlier, 5-FU is the mainstay of cancer therapy in clinics. Reports have shown that treatment with 5-FU can significantly alter the composition of gut microbiota, and the toxicity and adverse effects of 5-FU may partly be attributed to the changes in the gut microbial community and their unique physiological processes (Li et al., 2017; Yuan et al., 2018). One report showed that the side effects of 5-FU could be reduced by depleting the gut microbiota with antibiotics (Hamouda et al., 2017). However, another report demonstrated that the use of antibiotics could reduce the efficacy of 5-FU (Yuan et al., 2018). *Fitzpatrick* et al. reported that *E. coli* has the potential to improve the efficacy of 5-FU due to its ability to synthesize pyridoxal phosphate (PLP) and convert PLP to Vitamin B6 (Fitzpatrick et al., 2007).

Cyclophosphamide is another common chemotherapeutic agent for cancer treatment in clinics. A report showed that cyclophosphamide could induce the translocation of *Enterococcus hirae* and *Lactobacillus* spp. into the spleen and the mesenteric lymph nodes, and then lead to the anti-cancer effect via by stimulating T helper 17 (Th17) cells. Conversely, the antibiotics specific for gram-positive bacteria could reduce the effect of cyclophosphamide by decreasing the accumulation of TH17 cells in the spleen and reducing the infiltration of CD3+ T cells and Th1 cells (Vétizou et al., 2015). Furthermore, another report showed that *E. hirae* could improve the efficacy of cyclophosphamide due to inducing Th17 cells recruitment, however, *Barnesiella intestinihominis* could enhance the response of poly-functional Th1 and Tc1 cells to improve the drug efficacy (Daillère et al., 2016).

It is worth noticing that immunotherapy targeting checkpoint inhibitors are being approved for various kinds of cancer therapy. Monoclonal antibodies, including PD-1, PD-L1, and CTLA4 are proven to inhibit cancer progression and prolong the median survival time of patients (Badgeley et al., 2021). And more and more clinical evidence showed that GM composition was involved in the efficacy of the immune checkpoint inhibitors. A study used different sources of mice, from Jackson Laboratory and Taconic Farms to model melanoma mice, and the results showed that the mice from Taconic Farms displayed significant cancer progression compared to mice from the Jackson Laboratory. The authors found that *Bifidobacterium* was significantly accumulated in the mice from the Jackson Laboratory. Moreover, the combination of PDL-1 antibody and *Bifidobacterium* showed a better effect in inhibiting cancer development in mice bearing melanoma (Sivan et al., 2015). Fecal sample sequencing from the PD-1 and PD-L1 therapy responders showed that there was a significant difference of GM composition between the therapy responders and non-responders, for instance, the higher relative abundance of *A. muciniphila*, *Alistipes* spp., *Eubacterium* spp., and *Ruminococcus* spp. were existed in the responders (Matson et al., 2018). Another report also showed that the PD-1 therapy responders showed the higher relative abundance of *Faecalibacterium* species, while the lower relative abundance of *B. thetaiotaomicron* and *E. coli* when compared to the non-responders (Gopalakrishnan et al., 2018). Furthermore, certain studies also showed that when the mice was pre-treated with antibiotics cocktail, it significantly reduced the efficacy of PD-1, PD-L1, and CTLA4 immune checkpoint inhibitors (Vétizou et al., 2015; Routy et al., 2018).

### 3.4 GM can minimize NRB of antihypertensive drugs

Hypertension is a well-established risk factor that is closely linked to cardiovascular disease and is a major contributor to high mortality rates worldwide. The occurrence and development of hypertension are aggravated by both genetic and unhealthy lifestyle factors (Mills et al., 2016; Xiong et al., 2021). In recent decades, accumulating evidence has shown that GM also plays a significant role in hypertension. Dysbiosis of GM, alteration of GM metabolites, and an increase in potentially pathogenic bacteria in the gut can trigger the pathogenesis of hypertension (Santisteban et al., 2017; Wilck et al., 2017; Toral et al., 2019). Furthermore, there is growing evidence that GM can significantly impact the therapeutic effects of most antihypertensive drugs (Zimmermann et al., 2019; Robles-Vera et al., 2020).

As previously mentioned, nifedipine is a common therapeutic agent for hypertension in clinics. A study compared the effect of gut microbiota (GM) from rats fed at different latitudes on metabolizing nifedipine, and the results showed that GM could significantly affect the metabolism of nifedipine to oxidized nifedipine (Zhang et al., 2018). In addition, another report showed that a probiotic, *Lactobacillus casei*, could affect the intestinal absorption of nifedipine. The serum concentration of nifedipine in rats treated with *L. casei* was increased by 40% compared to the control group (Kato et al., 2007). In the case of amlodipine, the combination of antibiotics and amlodipine upregulated the serum concentration of amlodipine in rats treated with ampicillin (Yoo et al., 2016). Moreover, a recent study showed that depleting GM using antibiotics in captopril-resistant rats significantly improved the anti-hypertensive effect of captopril (Kyoung and Yang, 2022), indicating that manipulating GM composition could be an effective strategy for overcoming drug resistance. These results suggest that GM can significantly affect the absorption of anti-hypertensive drugs, which could be potentially leveraged to minimize non-responsiveness to treatment.

### 3.5 GM can minimize NRB of anti-arthritic drugs

Rheumatoid arthritis (RA) is an autoimmune disorder that poses a significant threat to people’s health. Despite significant advances in the understanding of RA pathogenesis and therapies, the morbidity of RA continues to rise year by year. Various reports indicate that approximately 350 million people worldwide, and nearly 43 million people in the United States, are affected by RA (Pahwa et al., 2022).

Methotrexate (MTX) is a dihydrofolate reductase inhibitor and the primary therapeutic agent used for RA. However, reports indicate that up to 50% of RA patients do not respond clinically to MTX (Emery et al., 2008; Detert et al., 2013). MTX can be metabolized to MTX-PGs, which are associated with the drug response by folylpolyglutamate synthase. However, removal of glutamate entities from MTX-PGs by carboxypeptidase could reduce the efficacy of MTX. A related study showed that Pseudomonas species is an important carboxypeptidase producer, which can potentially affect the clinical response of MTX (Levy and Goldman, 1967). In a study that enrolled new-onset RA patients who were MTX-naive, the relationship between GM and drug response was examined, and the results showed a significant correlation between GM composition and drug response, including orthologs related to purine and MTX metabolism. Furthermore, the remaining level of MTX after *ex vivo* incubation with distal gut samples from pretreatment RA patients was significantly related to clinical response, suggesting a possible effect of GM composition on MTX metabolism and treatment outcomes (Artacho et al., 2021).

Recently, Gupta et al. (2021) conducted a retrospective, observational cohort study on patients diagnosed with RA between 1988 and 2014. The authors used whole metagenome shotgun sequencing for the fecal samples to compare the GM composition between patients with or without minimum clinically important improvement (MCII). And the results showed that there was significantly different GM composition between the RA patients who eventually displayed MCII and those who did not. Patients who achieved clinical improvement had higher alpha diversity in GM composition at both baseline and follow-up visits. On the time-point of baseline, the taxa including Selenomonadales, Prevotellaceae, *Coprococcus*, *Bacteroides* sp., and *Bilophila* sp. were markedly increased in the MCII+ group, while *Eubacterium* sp. was accumulated in the MCII–group. However, on the time-point of follow-up, the taxa including Gammaproteobacteria, *Oscillibacter*, *Veillonella*, and *Bacteroides vulgatus* were higher in the MCII+ group. While the taxa including *Coprococcus*, *Ruminococcus*, *Anaerotruncus colihominis*, and *Oscillibacter* sp. were higher in the MCII− group. The authors suggested that the difference of GM composition could reflect MCII in RA patients in clinics. In summary, modifying GM composition to enhance clinical outcomes may hold promise as a future treatment for RA (Gupta et al., 2021). The effect of GM improving the drugs’ efficacy via minimizing NRB was shown in Table 2.

**Table 2:** GM minimizes NRB of drugs.

GM	Drugs	Category	Effects	Ref.
Probiotic cocktails including *L. acidophilus*, *B. bifidum*, and *B. longum*	Selenium	A clinical trial of AD patients	Probiotic cocktails plus selenium could significantly increase the Mini-Mental State Examination scores, and down-regulation of serum triglycerides, LDL, and total cholesterol when compared to the selenium and placebo groups	Tamtaji et al. (2019)
*L. rhamnosus*	Curcumin	Scopolamine-induced dementia mice model	*L. rhamnosus* plus curcumin showed a better effect on memory and cognitive improvement, and a prominent increase of antioxidant enzymes	Patel et al. (2020)
*Lactiplantibacillus plantarum* PS128	Levodopa	6-hydroxydopamine-inducing PD rat mode	*L. plantarum* PS128 ameliorate exaggerated M1 beta power spectral density and exhibit add-on therapeutic effects in combination with levodopa	Ma et al. (2021)
*Lactobacillus*	Metformin and Montelukast	Streptozotocin-inducing diabetes rat model	*Lactobacillus* combined with metformin and montelukast could significantly reverse the testicular and liver damage, as well as reduce the oxidative, inflammatory, and apoptotic activities caused by streptozotocin	El-Baz et al. (2021)
*E. coli* strains	5-fluorouracil	*C. elegans*	*E. coli* could enhance the biosynthesis of pyridoxal-5-phosphate to improve the effect of5-fluorouracil	Scott et al. (2017)
*Enterococcus hirae*				
*Barnesiella intestinihominis*	Cloposphamide	C57BL/6 mice	*E. hirae* could improve the efficacy of cyclophosphamide by inducing Th17 cells recruitment. *B. intestinihominis* could enhance the response of poly-functional Th1 and Tc1 cells to improve the efficacy of cyclophosphamide	Daillère et al. (2016)
*A. muciniphila*, *Ruminococcus*, *Faecalibacterium*	PD-1 and PD-L1 inhibitors	Mice bearing melanoma model	*A. muciniphila, Ruminococcus, and Faecalibacterium* were accumulated in the mice with responding of PD-1/PD-L1 inhibitors	Sivan et al. (2015) and Matson et al. (2018)
*L. casei*	Nifedipine	Rats model	The serum concertration of nefedipine in rats treated with *L. casei* was increased by 40% when compared to the mice in the control group	Kato et al. (2007)
*Pseudomonas* species	Methotrexate	Human fecal samples	*Pseudomonas* species is an important carboxypeptidase-producer that could removal of glutamate entities from MTX-PGs and reduce the effect of methotrexate	Levy and Goldman (1967)

## 4 Recommendation for NRB of drug through intervention

### 4.1 Administration of probiotics improves drug efficacy

*Bifidobacterium, Lactobacillus, Lactococcus,* and SCFA-producing bacteria have been shown to possess many beneficial properties for the host (Tavares et al., 2020; Abbasi et al., 2021; Cheng et al., 2022; He et al., 2022). Probiotics have also been demonstrated to minimize NRB of drugs and improve their efficacy. For example, *L. casei* gavage significantly increased the absorption of nifedipine when compared to the control group (without *L. casei*) (Kato et al., 2007). Another study showed that administration of *L. casei* significantly increased the antiprotozoal effect of albendazole in both severity and duration of Giardia infection (Shukla et al., 2013). Chemotherapy for cancer patients usually causes significant adverse effects and dysbiosis of the GM, which limits the efficacy of drugs. Several studies have found that administration of probiotics or probiotic cocktails can help to reduce the adverse effects and reinstate GM dysbiosis (Alexander et al., 2017; Legesse Bedada et al., 2020; Xia et al., 2021).

There are certain points that need to be considered before probiotic application. First, it is important to ensure that sufficient live bacteria can be implanted in the host gastrointestinal tract (Feng et al., 2020). The strong pH of the stomach can kill the ingested bacteria, but selecting acid- and bile-resistant strains and using protection technology like microencapsulation can solve this problem (Iravani et al., 2015; He et al., 2022). Additionally, some probiotics, such as *Bifidobacterium*, *Faecalibacterium prausnitzii*, and *Lactobacillus*, are anaerobic and can lose their effect when exposed to oxygen (Ladero and Sánchez, 2017; He et al., 2022). Therefore, selecting oxygen-resistant and hydrogen-peroxide-resistant variants of *Bifidobacterium* using adaptive evolution strategy can be useful (Mozzetti et al., 2010; Zhang et al., 2020a). This information shows that the survival and metabolism models of probiotics can possibly change under specific stress, and maintaining stable strains of probiotics is also a crucial aspect of investigating their effects. Verification of the safety and effects of probiotics should be based on the strain level. For example, various strains, including *B. animalis* subsp. lactis Qq08, *B. animalis* subsp. lactis BB-12, and *B. animalis* subsp. lactis INL1, belong to the species of *B. animalis* and may possess different growth and metabolism characteristics that need further clarification (Li et al., 2010; He et al., 2022).

Manipulating the gut microbiome with probiotics to improve the efficacy of a specific drug seems like a useful option for personalized medication. For instance, *Akkermansia muciniphila* and *Bifidobacterium adolescentis* have been demonstrated to have positive benefits in Type 2 diabetic animals when added to a diet (Lam et al., 2019). Studies to characterize the gut microbiome in terms of mechanistic insights into how they improve drug efficacy in various health conditions would be crucial for the development of innovative medications and possibly customized treatments.

### 4.2 The role of prebiotics in minimizing NRB and improving the drug efficacy

Prebiotics refer to non-digestible food components, including fibers, chitin, and polysaccharides, that selectively promote the growth of beneficial bacteria and positively affect the host’s physiology (Al-Sheraji et al., 2013; Xia et al., 2020; Li et al., 2022; Yin et al., 2022). Plants, food, and ethnic herbal medicines are the primary sources of prebiotics. Several studies have reported that prebiotics stimulate the growth of beneficial bacteria, such as Bifidobacterium and *Lactobacillus*, while inhibiting the growth of potential pathogens such as *Helicobacter*, *Shigella*, and Fusobacteria. In addition, prebiotics can also ameliorate the detrimental effects of drugs.

Recent studies have found that prebiotic polysaccharides, such as those derived from *Poria cocos* and *Grifola frondosa*, can significantly reinstate the gut microenvironment that is damaged by chemotherapy drugs such as 5-Fu (Fluorouracil) and improve their anticancer effects (Mao et al., 2018; Yin et al., 2022). These studies also showed that the growth of probiotic species, such as *B. animalis* and *L. jonsonii*, stimulated by prebiotics contributed to the improvement of the efficacy of 5-FU.

Other studies have found that prebiotics such as oligofructose (OFS), mannan-oligosaccharides (MOS), and inulin can improve the effects of metformin on ameliorating insulin resistance and glucose tolerance, repairing islet and hepatic histology, and reducing fat mass, lactate, and phosphatidylcholine levels (Zheng et al., 2018; Lam et al., 2019; Li et al., 2020). These findings suggest that prebiotics can have a mild impact on the gut flora, which could directly or indirectly affect the host’s response to various medications/drugs. Therefore, it would be intriguing to investigate whether and how prebiotics can aid in improving various health complications in humans, eliminating side effects, and/or increasing drug efficacy.

### 4.3 FMT minimized NRB and improved the efficacy of drug

FMT is a method for directly transplanting the microbiota from a healthy donor into the intestinal tract of a recipient to normalize the composition of their microbiota and gain therapeutic benefits. In 2013, the first randomized controlled trial of FMT demonstrated that duodenal infusion of donor feces in recurrent *Clostridium difficile* infection patients had better efficacy in resolving symptoms than antibiotics used alone (van Nood et al., 2013). Since then, numerous associated research studies have demonstrated that FMT can significantly benefit gut-related diseases (Weingarden and Vaughn, 2017; Fong et al., 2020; Zhang et al., 2020b). Today, the application of FMT has rapidly expanded from gastrointestinal disorders to extra-gastrointestinal diseases (Ooijevaar et al., 2019; Wang et al., 2019; Vendrik et al., 2020). For example, On April 26, 2023, the US Food and Drug Administration approved the first oral microbiota-based product Vowst that consists of a highly purified collection of about 50 species of Firmicutes spores to prevent recurrent *C. difficile* infection, and the Vowst was identified as the restoration of colonization resistance in the gut and the metabolic competition between the microbiome therapy and *C. difficile* (Jain et al., 2023). Moreover, a single-arm clinical trial investigated the effect of FMT on intestinal graft-versus-host disease in allogeneic hematopoietic cell transplantation recipients, and the results showed that FMT recipients had high gut microbial alpha diversity and an increased abundance of butyrate-producing bacteria, while reducing the need for immunosuppressive drug therapy (van Lier et al., 2020). A pilot randomized controlled study exploring FMT in Crohn’s disease showed that FMT could help to improve the clinical remission rate of patients treated with corticosteroid (Sokol et al., 2020). Several recent studies have also shown that FMT can significantly enhance the efficacy of immune checkpoint inhibitors on cancer progression. A clinical trial evaluated the safety and efficacy of responder-derived FMT together with anti–PD-1 in PD-1–refractory melanoma patients. The results showed that the FMT responders exhibited an increase in gut microbiota abundance related to a response to anti–PD-1, an increase in CD8+ T cell activation, and a decrease in IL-8 expressing myeloid cells (Davar et al., 2021). Additionally, a phase I clinical trial to evaluate FMT for patients with anti-PD-1-refractory metastatic melanoma demonstrated that FMT could change immune cell infiltration and gene expression in both gut lamina propria and the tumor microenvironment, leading to improved anti-cancer effects of the PD-1 inhibitor (Baruch et al., 2021). However, several studies also showed that the negative or no significant difference results of FMT in irritable bowel disease (Ianiro et al., 2019; Myneedu et al., 2019; Xu et al., 2019). The reasons for the contradictory effects of FMT probably include both FMT excipients and autologous FMT, research showed that capsule excipients may increase the magnitude of placebo effects through psychological and neurobiological mechanisms (Shah and Pimentel, 2014). Moreover, the resource of FMT, for instance, luminal and mucosal source in the upper and lower GI tract have significant differences of GM composition, is another factor that impact the effect of FMT (Zmora et al., 2018; Xu et al., 2019). Additionally, laxatives used for bowel preparation before FMT may alter gut microbiota (Tropini et al., 2018; Zmora et al., 2018), and glycerol used as a cryoprotectant (Cleusix et al., 2008), may also impact GM composition to bias the effects of FMT.

Despite the potential prospects of FMT in improving drug efficacy, several issues must be addressed. First is the selection of FMT donors. Strict selection protocols can reduce the adverse effects of FMT, particularly the infection risk from donor to recipient. Some studies have recommended that donors undergo blood and stool examinations within 4 weeks before donation (Paramsothy et al., 2015; Cammarota et al., 2017; Wang et al., 2019). Most clinical trials and systematic reviews have shown that adverse events, such as diarrhea, abdominal discomfort, constipation, and low-grade fever, are typically transiently noted after FMT (Brandt et al., 2012; Cammarota et al., 2014; Rossen et al., 2015). Secondly, the quality control of the donors’ samples is essential. GM composition can be affected by many environmental factors. It is important to ensure that feces collected from donors at different time points or batches have a similar GM composition. Additionally, collection and storage conditions of samples are critical due to the anaerobic properties and temperature sensitivity of certain gut microbiota. Finally, the recipient’s immunity is a crucial factor that can affect the efficacy of FMT. Many pre- and clinical studies have shown that some recipients do not respond well to FMT due to the recipient’s immune system inhibiting the growth of GM (van Nood et al., 2013; Wang et al., 2019; Baruch et al., 2021).

**Figure 3:**
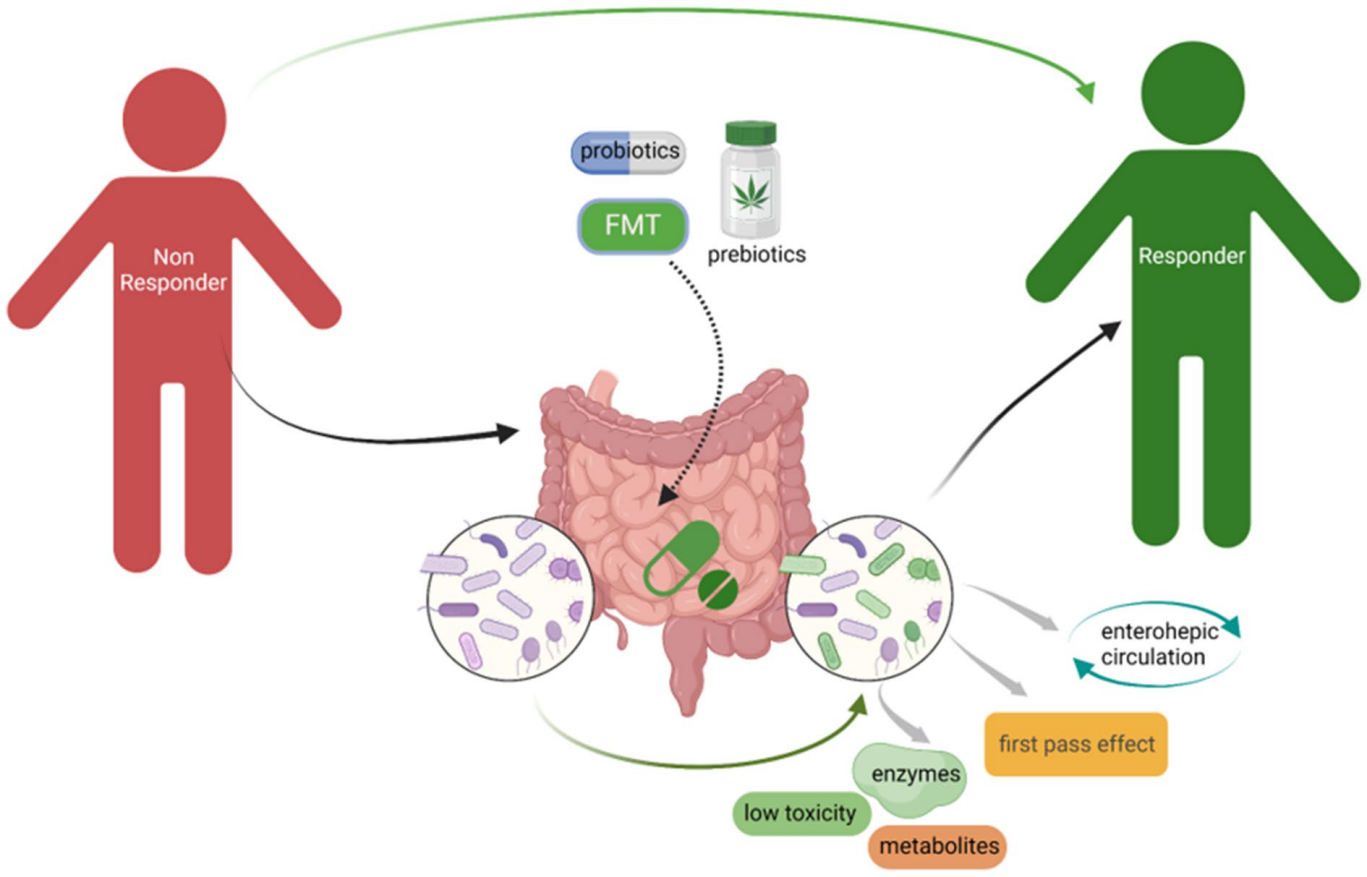
Administration of probiotics, prebiotics, as well as accepting FMT could minimize NRB to improve the drugs efficacy. The change of GM composition especially increased the beneficial bacteria, for instance, *Bifidobacterium* and *Lactobacillus*, could significantly limit NRB through the microbial derived enzymes that could affect the drugs availability.

In Parts 4.1 to 4.3, we highlighted that administration of prebiotics and probiotics, as well as accepting FMT, which could change GM composition probably one of the potential strategies to minimize NRB to improve the drug efficacy (Figure 3). There is another important point to highlight that GM composition is remarkably impacted by individual diet. Dietary supplements with fiber, chitin, and polysaccharides could significantly change GM composition, especially increasing the relative abundance of Bifidobacterium, *Lactobacillus*, and short-chain fatty acids-producing bacteria, however, reducing potential pathogens including Helicobacter, Shigella, and sulfate-reducing bacteria, which could benefit for the host gut microenvironment and help to improve the drug efficacy and bioavailability (Khan I. et al., 2018; Xia et al., 2020; Huang et al., 2022; Yin et al., 2022). A recent study focusing on the relationship between diet–microbiota interactions and type 2 diabetes and coronary heart disease, from which results showed that 17 microbial species that related with higher tryptophan intake and higher indolepropionate concentrations were observed in the group with higher fiber intake. Dietary fiber intake could increase the microbial metabolites indolepropionate and tryptophan that could help to reduce the risk of diabetes and coronary heart disease (Hu et al., 2023). Although we could find many pre-clinical studies in this field, more clinical studies still need to be performed to verify the effect of GM on the impact of drugs availability and efficacy.

### 4.4 Understanding and minimizing NRB with the help of advances in multi-omics technologies, big-data, synthetic and engineered microbial communities

The human gut is home to a wide variety of microbes, making it difficult to determine how each microbe within the microbiome interacts with the drug of interest inside the host. Moreover, in the tripartite interaction between the host, drug, and microbiome, both the host and the microbiome have distinctive roles in influencing drug efficacy. Therefore, it becomes very difficult to decipher how only the microbiome part interacts with the drug, excluding the host involvement. The use of bacterial consortia or individual culture-based techniques seems a plausible option in this scenario to dissect the drug-microbiome interactions. However, it will be challenging to comprehend microbiome-drug interactions due to a number of constraints, such as the fact that only a tiny portion of the total gut microbiota is culturable (Martín et al., 2014, 2015; Boesmans et al., 2018; Depommier et al., 2019; Vázquez-Castellanos et al., 2019). This further suggests that utilizing the whole gut microbiome *in vitro* will not be possible for elucidating such interaction studies in understanding microbiome-mediated NRB. The idea of utilizing a defined and controlled synthetic bacterial community (SynCom), comprising a small number of culturable bacterial members of the gut, could partially address this issue. Additionally, SynCom could possibly mimic the structure and functions of the original gut microbiome (Vázquez-Castellanos et al., 2019). The SynCom technique has the significant advantage that it allows us to manage this community to achieve desired functionalities, such as probiotic characteristics, by the addition, removal, or changing any strain (s) of interest. Such adjustments can also be made at the genetic levels of the strain; for instance, specific SynCom member microbe functions can be eliminated or enhanced utilizing gene silencing or increased expression, respectively. Thus even engineered strains can be utilized to be part of the SynCom. The member strains of SynCom are culturable, which makes them suitable for using reductionist methods to analyze the structural complexity and functions connected with the microbiota. When examining drug-microbiome interactions *in vitro*, SynCom techniques may be very helpful, where such interactions could finally be narrowed down to specific members of the SynCom. Additionally, SynCom could be utilized in germ-free organisms to determine the tripartite interactions between host, microbiome, and the drug (Vázquez-Castellanos et al., 2019).

Host-microbiome interactions have been largely clarified using recent technological advancements. These include the use of multi-omics such as metagenomics, metatranscriptomics, metaproteomics, and metabolomics (Roy et al., 2019). Additionally, artificial intelligence and the availability of big data have further helped in drawing useful conclusions from various association studies. The Human Microbiome Project, The American Gut, The European Microbiome Project, and The Asian Microbiome Project are a few of these big data initiatives that merit mentioning because they have generated enormous amounts of data and significantly advanced our understanding of host–microbiome interactions (Khan et al., 2022). This has also made it easier to provide gut bacterial strain biobanks with a variety of bio resources and have standardized the procedures for structural and functional profiling of the microbiota associated with the host (Turnbaugh et al., 2007; McDonald et al., 2018). The idea of the core microorganisms, which contends that some bacterial groups are essential for preserving the structure and functionality of the unique gut microbial community, is an important feature of host-microbiome interaction that has so far been understood (Tap et al., 2009; Kearney et al., 2018; Ju et al., 2019). Therefore, an integrated approach based on the knowledge, data, and resources from the above-mentioned big projects backed with current technological advances could be utilized for building customized SynCom. These SynCom will comprise core microbes along with some other important members of the community, which could then be utilized for dissecting the microbiome-drug interaction both *in-vitro* and *in-vivo*.

Additionally, the most recent developments in artificial intelligence (AI), such as machine learning algorithms, could be used to analyze the fundamental relationships between the host microbiome (Namkung, 2020) and an array of available drugs. Our understanding of how the gut microbiome increases or decreases the efficacy of various drugs could be further improved by AI-based big data comparative studies of the gut microbiota in groups with diverse drug usage. Finally, information from such AI-based data could be used to find and select specific gut bacterial genera involved in enhancing drug efficacy. These selected strains, based on big data, could then be utilized to optimize and modulate SynCom for a variety of medical applications.

Moreover, Fecal Microbiome Transplants (FMT) from individuals with a better response to drugs of interest is another option to enhance drug efficacy in individuals who do not respond well. However, conventional FMT has various limitations, including but not limited to the transfer of pathogenic microorganisms, transient results, lack of reproducibility, and high donor screening costs (Khan et al., 2022). Customized SynCom-based microbiota transplants, which can potentially act as a representative of the structure and function of the original gut microbiome, seem like a better alternative to conventional FMT. Thus, SynCom-based transplants of individuals with a better response to drugs of interest can be utilized to enhance drug efficacy. As comprising defined bacterial members, Syncom-based transplants are a relatively safe, reproducible, and economical option in drug-microbiome interaction-related studies.

Since the majority of the gut microbiome is unculturable, and among those that are culturable, the majority will be microorganisms responsible for interacting with the drug and thereby increasing and decreasing its efficacy. Now the question arises, how can the probiotic potentials of those unculturable members be utilized to enhance the drug efficacy in individuals with a low response to that drug? One possible solution could be the utilization of functional bioprospecting metagenomics. Utilizing functional bioprospecting metagenomics, one can capture the genetic potentials of the unculturable organisms in a heterologous host (Khan et al., 2016; Khan R. et al., 2018; Kim et al., 2020). The heterologous host is capable of expressing the genetic potential on a recombinant plasmid vector and can thus be used to confirm whether the genetic component captured can replicate the function of the original gene content in the heterologous host (Khan et al., 2015, 2016; Khan R. et al., 2018). Once confirmed, the heterologous host itself can be utilized as an engineered probiotic or the recombinant plasmid carrying the genetic element with the potential to enhance drug efficacy can be transformed to other culturable beneficial members of the gut and can be utilized to enhance the efficacy of the drug in question. Thus, functional bioprospecting metagenomics can be utilized to design engineered probiotic strains with the potential to modulate drug efficacy inside the host.

A more natural way of modulating the gut microbiome to have a desired outcome regarding the enhanced efficacy of a drug is the use of bacteriophages. Single bacteriophage or a consortium of various bacteriophages targeting the bacterial taxa of interest could be utilized to modulate the structure of the gut microbiome (Rasmussen et al., 2020). However, to do that, one would initially need to develop various bacteriophage biobanks that selectively target desired bacteria involved in either deactivating the drug or decreasing the efficacy of the drug. Then, various bacteriophage cocktails can be designed and customized according to the medical condition and the drug of interest to see how such cocktails affect their efficacy. For instance, bacteria involved in the inactivation of drugs can be targeted and selectively eliminated with the help of bacteriophages in this regard.

**Figure 4:**
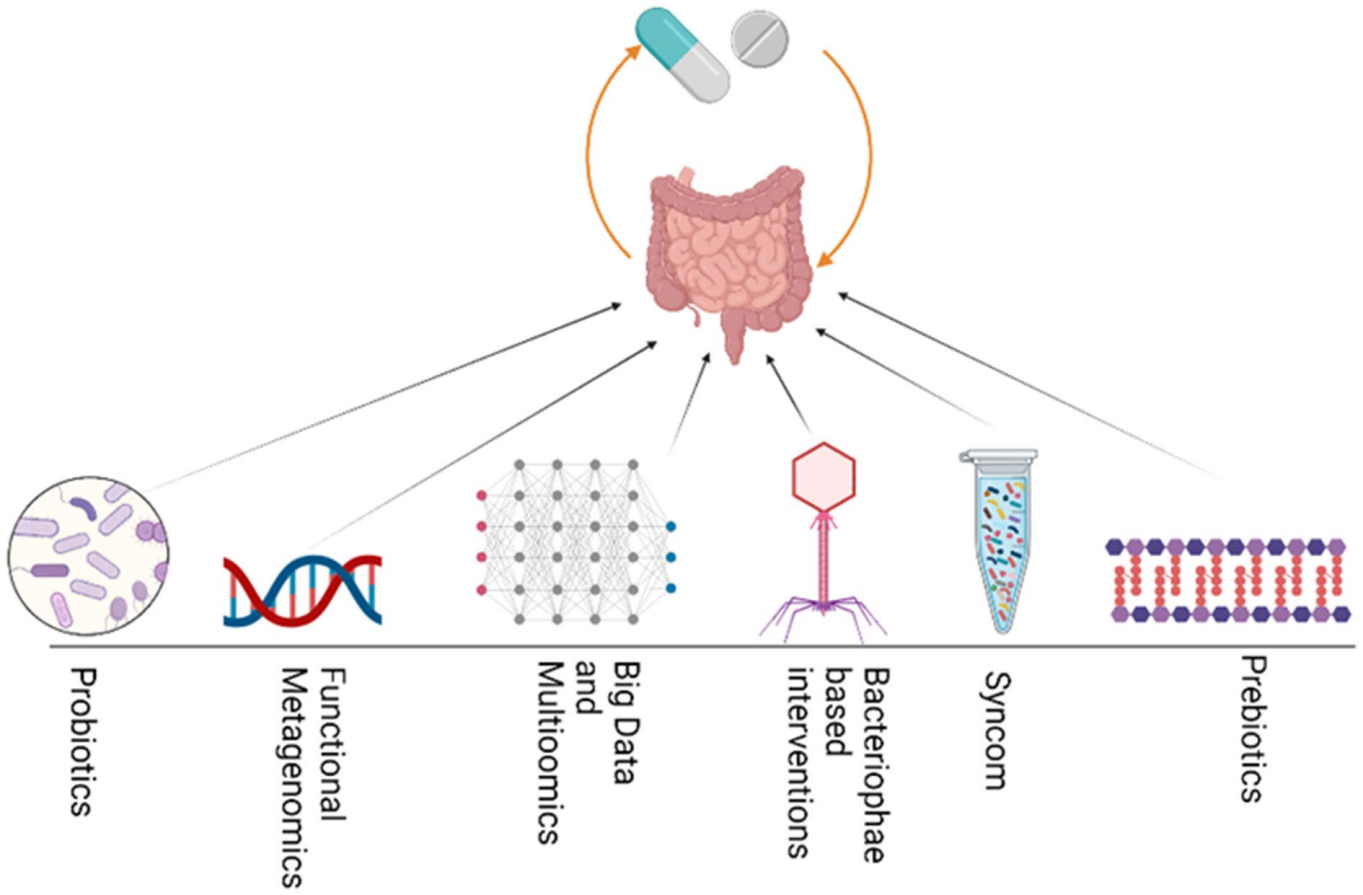
Various integrative approaches could be utilized to modulate GM and thus enhance drug efficacy. These strategies include the use of Probiotics, functional bioprospecting metagenomics, Big data mining and multiomics, bacteriophage, Synthetic microbial communities and prebiotics.

Various fields of study aim to unravel the bipartite interactions and associations related to drugs, and can be integrated to enhance our understanding of how drugs affect individuals. For example, pharmacometagenomics, pharmacomicrobiomics, pharmaco metabolomics, pharmacoproteomics, pharmacotranscriptomics, and pharmacoepigenomics are important fields that can be integrated to study how drugs interact with microbiomes and both the host and microbiome-associated metabolites, genes, proteins, and epigenetic modifications. By studying these interactions, we can gain insight into how different individuals respond to different drugs. Integrating such multipartite interactions can help control and minimize non-response bias (NRB). Additionally, by utilizing knowledge gained from integrative approaches, researchers may be able to develop more personalized and effective drug therapies tailored to the unique characteristics of each patient’s microbiome and genetic makeup. Ultimately, these approaches can lead to improved health outcomes in patients and a more efficient use of healthcare resources. Collectively, various integrative approaches (Figure 4) can be used to manipulate and modulate the gut microbiome in specific medical conditions to reduce NRB and improve the efficacy of a specific drug, thus achieving desired outcomes.

### 4.5 Discrepancies and the challenge of correlation versus causation in microbiome studies

Contemporary research has delved into the intricate relationship between the GM, health, and disease, yielding compelling but occasionally conflicting insights. Varied microbial profiles have resulted in disparities among populations, influenced by dietary habits and ethnic origins. This underscores the significance of factoring in geographical and lifestyle variables in microbiome investigations (Yatsunenko et al., 2012). Nonetheless, it’s important to recognize that correlational observations often fall short of establishing causation. At times, it can be challenging to discern whether a particular phenotype is a consequence of the observed variation or if it is indeed the root cause of that variation (Ebrahim and Davey Smith, 2008). For instance, while earlier research has established connections between specific microbial species and health conditions, recent studies involving diverse populations have at times yielded conflicting findings (Zhernakova et al., 2016). The task of interpretation is further compounded by variations in methodology across investigations, encompassing disparities in DNA extraction techniques and data processing methods (Knight et al., 2018). As the field of research evolves, it becomes imperative to embrace a holistic approach that not only considers the intricate interactions within the complete microbial ecosystem but also takes into consideration other influential factors such as host genetics and dietary choices (Clemente et al., 2012). Given that the study of the microbiome is a burgeoning field of science, it often entails operating at the cutting edge of an evolving discipline. This inevitably brings with it a certain margin of error, which is subsequently refined and minimized through the application of advanced methods and tools.

## 5 Conclusion

In summary, this manuscript discusses the impact of GM on drug metabolism and effectiveness, specifically focusing on drugs like digoxin, diltiazem, and nifedipine. In addition, this study is highlighting specific genes encoded by GM that can metabolize drugs and alter their bioactivity or toxicity. The manuscript also summarized the findings of studies that investigated the promotion of beneficial bacteria growth after administering medicine to hosts. In contrast, Sousa et al., Turnbaugh et al., J. Gilbert, Kim & Cho discuss various aspects of gut microbiota, including their composition, function, and impact on health and disease. They explore the potential of GM modulation as a therapeutic approach for various conditions. These works do not specifically focus on the impact of GM on drug metabolism and effectiveness.

## Author contributions

GH: Investigation, Writing – original draft, Writing – review & editing. RK: Investigation, Writing – original draft. YZ: Data curation, Investigation, Writing – review & editing. P-CL: Data curation, Validation, Writing – review & editing. QL: Supervision, Validation, Writing – review & editing. IK: Data curation, Supervision, Validation, Writing – review & editing.
